# Novel image analyser-assisted morphometric methodology offer unique opportunity for selection of embryos with potential for implantation

**DOI:** 10.1186/s12884-023-06025-2

**Published:** 2023-09-28

**Authors:** Y Alhelou, M Hamdan, N Razali, NAM Adenan, J Ali

**Affiliations:** 1 FAKIH IVF, Sh Haza Bin Zayed st, Abu Dhabi, United Arab Emirates; 2 Department of Obstetrics and Gynaecology, Universiti Malaya, Kuala Lumpur, Malaysia; 3 IVF Department, Maternity and Children Hospital, Dammam, Saudi Arabia

**Keywords:** Embryo morphometry, Embryo selection, Time-lapse, Perivitelline Space, Zona Pellucida

## Abstract

**Background:**

Previous studies looked into the connections between pregnancy and the Zona Pellucida (ZP) thickness and Zona Pellucida Thickness Variation (ZPTV), as well as the embryo’s radius, circumference, perimeter and global symmetry. However, no research has linked embryo implantation and pregnancy to the percentage of ZP thinning, the reduction in ooplasm volume, and the increase in perivitelline space (PVS) volume. Our objective is to correlate the percentage of ZP thinning, the percentage of ooplasm volume shrinkage and the percentage of PVS increase to the implantation. These data will be used for embryo selection as well as it can be put into a software that will assist embryo selection.

**Materials and methods:**

Retrospective study included 281 patients, all of them had 2 embryos transferred, 149 patients got pregnant with two gestation sacs and 132 patients did not get pregnant. All of the transferred embryos had the ZP thickness measured several times from time of ICSI till Embryo Transfer (ET), the ooplasm volume was calculated from time of ICSI till two Pronuclei (2PN) fading and the PVS was calculated from the ICSI time till the 2PN fading.

**Results:**

The first characteristic is the change in the average ZP thickness that decreased by 32.7% + 5.3% at 70 h for the implanted embryos (Group 1) versus 23.6% + 4.8% for non-implanted embryos (Group 2) p = 0.000. The second characteristic is the average reduction in the volume of the ooplasm which is 20.5% + 4.3% in Group 1 versus 15.1% + 5.2% in Group 2, p = 0.000. The third characteristic is the increase in the volume of the PVS which was 38.1% + 7.6% in Group 1 versus 31.6% + 9.7% in Group 2 p = 0.000.

**Conclusion:**

The implanted embryos showed higher percent of ZP thinning, higher percent of ooplasm reduction and higher percent of PVS increase.

## Background

Proper and accurate embryo selection in assisted reproduction treatment is a paramount factor in achieving pregnancy [1–3] in addition to maternal age factor [4]. The selection of viable embryos for transfer is essential for efficient treatment which in turn reduces multiple pregnancies rate that leads to maternal and neonatal complications [5, 6]. Such risks can be minimised by reducing the number of the embryos transferred [7]. Therefore, new embryo selection techniques are needed to improve selection of single best embryos without compromising the implantation and live birth rate.

To date, non-invasive techniques for embryo selection are still based on morphological assessment which analyse the number and symmetry of blastomeres, presence and percentage of fragmentation, multinucleation and thickness of the zona pellucida [8–12]. These techniques are vastly subjective and are considered insufficient [13] and are heavily influenced by the age of the woman.

Additionally, time lapse incubation is advantageous as it permits additional kinetic markers application for embryo selection [14–18] and enables the development of potentially useful algorithms that may facilitate selection of the potentially implantable embryo [19].

There are studies that have linked the oocytes and embryos morphometric characteristics to fertilisation [20] and implantation [18, 21–23] respectively. One study correlated pregnancy to the thickness of the zona pellucida and found that embryos with thinner ZP are more likely to implant with odd ration (OR) 0.774, the measurement of the embryo were estimated from the pictures of embryos taken immediately prior to transfer [23]. A second study correlated the area and the perimeter of the embryo, in relation to the radius of the circumference of the embryo, embryonic global symmetry, thickness of the zona pellucida, area and perimeter of the blastomeres, radius of the blastomeres and blastomeric global symmetry to pregnancy [21], these parameters were evaluated from the photographs taken immediately before embryo transfer. Paternot et al. linked embryo volume to pregnancy [18]. Palmstierna et al. and Gabrielsen et al. found a correlation between the zona pellucida thickness variance (ZPTV) and pregnancy [24, 25]. Garside et al. found that embryos with thinner zona pellucida have higher implantation rate [26].

The objective of this study is to investigate the changes in the thickness of the ZP, the contraction of the ooplasm and the increase in the perivitelline space and link the changes to the implanted and non-implanted embryos.

## Materials and methods

This retrospective study was conducted in a referral IVF center examining 281 patients with double embryo transfer of which 149 patients became pregnant with two gestation sacs and 132 patients did not get pregnant. The study was approved by the research and ethics committee at Fakih IVF, all methods were performed in accordance with the relevant guidelines and regulations.

### Oocyte retrieval

The study was performed between June 2016 and November 2016. All patients were childless couples that utilised their own oocytes. Women under 35 years of age with regular menstrual cycles, normal uterine ultrasounds, undergoing their first or second ICSI trial and a body mass index (BMI) between 18 and 30 kg/m^2^ and women that achieved twin pregnancy, and woman who did not achieve pregnancy after the treatment procedure were included. Women pregnant with single gestation sac, males with a total sperm count totalling less than 5 million, males over 50 years of age, patients with any uterine conditions, endocrinopathies or recurrent pregnancy loss, and patients undergoing treatment for any other medical condition were excluded. Controlled ovarian hyperstimulation was performed according to the standard method for short antagonist protocol using recombinant subcutaneous FSH (Gonal-F, Merck Serono, Dubai). FSH doses were regulated individually according to patients’ response. Finally, a dose of 250 micrograms choriogonadotropin alfa (Ovitrelle, Merck Serono, Dubai) was administrated subcutaneously when three follicles were 17 mm or larger. Ultrasound-guided oocyte retrieval was performed 36 h after choriogonadotropin alfa administration. Patients’ characteristics are given in Table 1.

**Table 1:** Patients’ Characteristics

Parameter	Group 1 Pregnant	Group 2 Non-Pregnant
No of cases	149	132
Age mean SD	30.1 3.6	29.9 3.9
BMI SD	24.0 3.4	25.3 3.4
FSH Baseline mIU/ml SD	6.5 1.7	6.7 1.8
E2b baseline SD	29.4 11.2	29.3 11.1
Mean FSH dose IU SD	2104 424	2129 409
Mean No of oocytes SD	9.9 3.3	9.3 3.3

### ICSI and oocyte incubation

Assisted hatching and biopsy were not conducted for patients included in this study. After follicle aspiration, oocytes were washed with Global w/HEPES media (Cooper Surgical Group, USA) then cultured in Global fertilisation medium (Cooper Surgical Group, USA). Oocytes were stripped of their cumulus cells after 1 h of oocyte retrieval in Hyaluronidase medium (80 IU/ml; Cooper Surgical, USA). All oocytes were inseminated by ICSI after 1 h of oocyte retrieval in global w/HEPES media under magnification of x400 using an inverted microscope (Olympus IX-73, Japan) and micromanipulator (Narishigie, Japan).

After ICSI insemination, oocytes were individually cultured in pre-equilibrated droplets of 25 µL of Global Total LP medium (Cooper Surgical Group, USA) overlaid with 1.4ml of mineral oil (LIGHTOIL, Cooper Surgical, USA) in an embryoscope dish (EmbryoSlide™, Vitrolife, Sweden). All embryos were incubated in a time lapse incubator (EmbryoScope™, Vitrolife, Sweden) at 37^o^C under atmosphere of 6.0% CO_2_, 6.0% O_2_ and 88% N_2_, and pH of 7.28 to 7.32. The EmbryoScope™ time lapse incubator is equipped with a microscope and a camera that is programmed to snap pictures of up to 72 embryos individually during the incubation period. The camera was set to take pictures at 5 different focal planes every 10 min. It has been proven that the EmbryoScope™ time lapse incubator is safe for human and animals’ embryos [14, 26–28].

The successful fertilisation and the quality of the embryos were video-recorded using the EmbryoViewer™ software attached to the Embryoscope (Vitrolife, Sweden). The embryo development video recordings were checked for fertilisation on day 1 between 16 and 18 h after ICSI. Day 2 observations were performed between 44 and 46 h after ICSI, day 3 observations between 66 and 68 h. The quality of the embryos was documented with specific reference to the number and symmetry of blastomeres, percentage fragmentation and multinucleation. Embryos were graded on a numerical scale from 1 to 4, where 1 denotes poor embryo quality and 4 indicates excellent embryo quality. Grade 4 was assigned to embryos if their blastomere shapes were even and there was less than 10% fragmentation within the embryo. Embryos were graded “3” when their blastomeres were uneven and of different sizes but limited to a variation of no more than 20% of each other and with 10–20% fragmentation. Embryos were graded “2” when blastomeres were uneven in size with differences in sizes of between 20 and 50% of each other and with 20–50% fragmentation. Embryos were graded “1” when their blastomeres had a more than 50% difference in size from one another and more than 50% fragmentation. Embryo assessments were performed by the same embryologist.

In addition to morphological evaluation, embryos were evaluated for morphokinetic parameter corresponding to the time taken to reach a specific cell stage between 1 cell and 9 cells. The time corresponding to one cell stage was annotated as t1, t2 for two observed cells, and so on. The duration of the second cell cycle (cc2) corresponding to the duration of the two-cell embryo phase calculated by subtracting t2 from t3, and cell division synchronisation (s2) corresponding to the time required for each blastomere to replicate and reach a successive cell cycle, were also recorded. s2 was calculated by subtracting t3 from t4. The morphokinetic algorithm described by Meseguer et al. [19] was used in the selection of embryos for transfer.

### Embryo transfer

Double embryo transfer was performed on day 3 under ultrasound guidance. Luteal phase support was performed with 400 mg of Cyclogest (Actavis, UK) administered rectally twice a day and Oestradiol valerate 2 mg (The white tablet of Progyluton, Bayer, Germany) tablet was administered twice a day. The β-hCG concentration was measured 12 days post embryo transfer and the clinical pregnancy was determined on the ultrasound detection of foetal heartbeat on week 7 following embryo transfer. All pregnancies were referred to the Obstetrics and Gynecology Department for follow up.

### Measurements

**Fig. 1:**
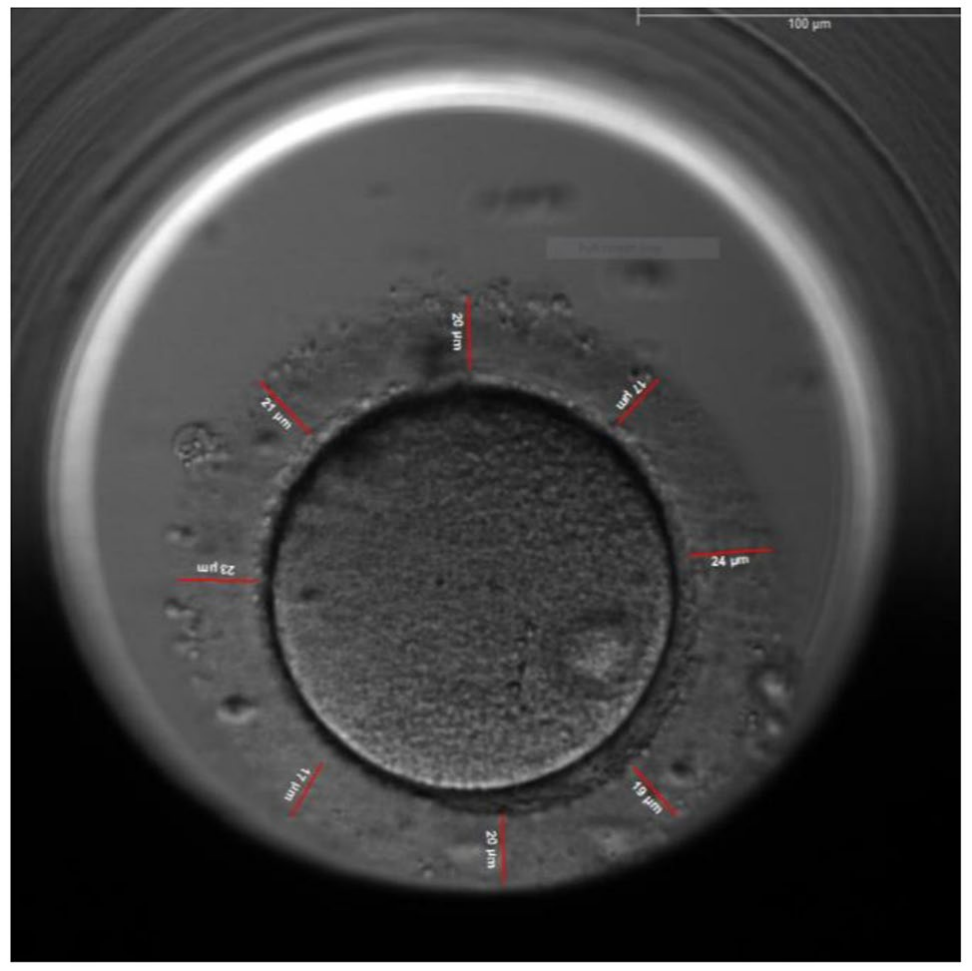
Measurement of the zona pellucida thickness at 8 different points

**Fig. 2:**
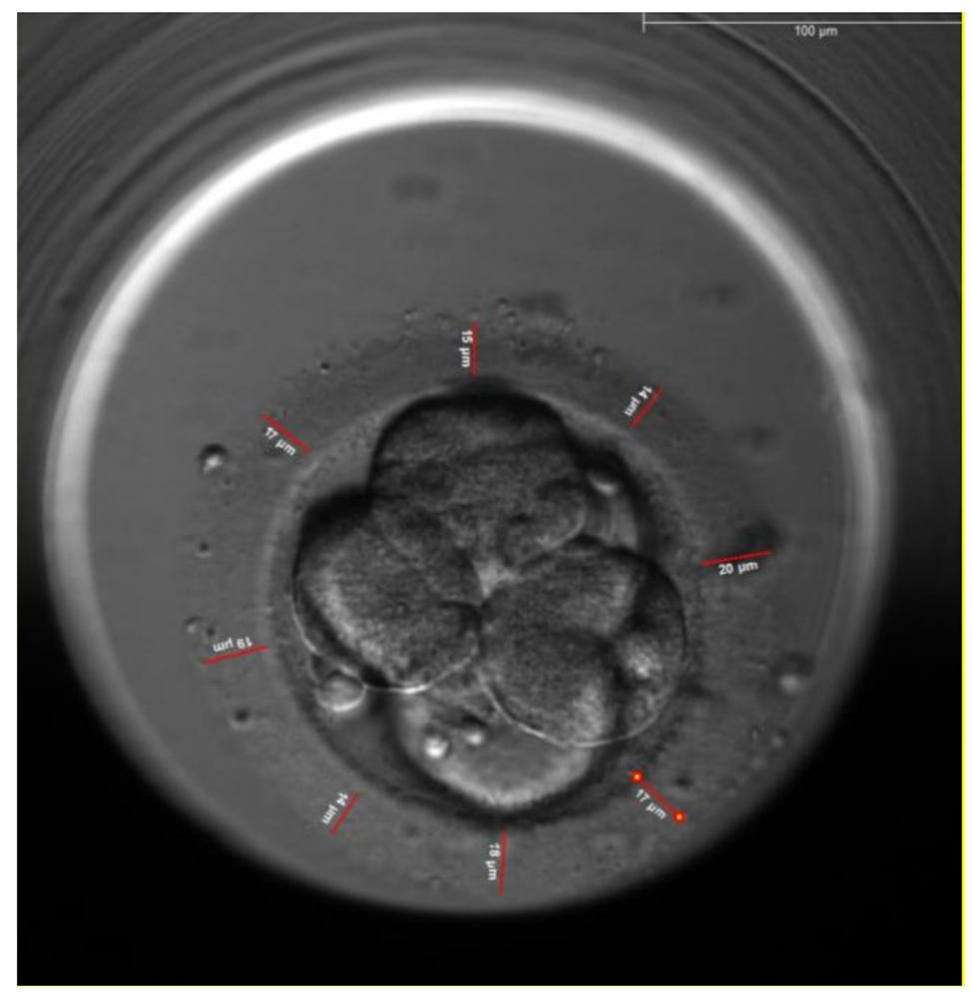
Measurement of the zona pellucida at 70 h after ICSI. The same oocyte in Fig. 1

The thickness of the zona pellucida was measured at 8 different points, the average thickness was calculated, the thickness of the ZP of each embryos was measured every 10 h interval starting from the time ICSI was performed (Fig. 1) till 70 h post ICSI (Fig. 2), the distance tool of the EmbryoViewer™ software was used for the measurement of the ZP thickness.

The reproducibility of measurements was confirmed by repeating the ZP measurements 20 times for 10 oocytes and calculating the standard deviation and coefficient of variation (Table 2).

**Table 2:** Reproducibility of measurements of ZP thickness

Oocyte	Mean µm	SD µm	CV (%)
1	19.11	0.13	0.67%
2	18.23	0.14	0.76%
3	20.34	0.16	0.81%
4	19.08	0.19	0.97%
5	18.78	0.13	0.70%
6	19.00	0.15	0.80%
7	19.24	0.17	0.91%
8	18.59	0.15	0.81%
9	17.69	0.13	0.73%
10	19.58	0.19	0.97%

**Fig. 3:**
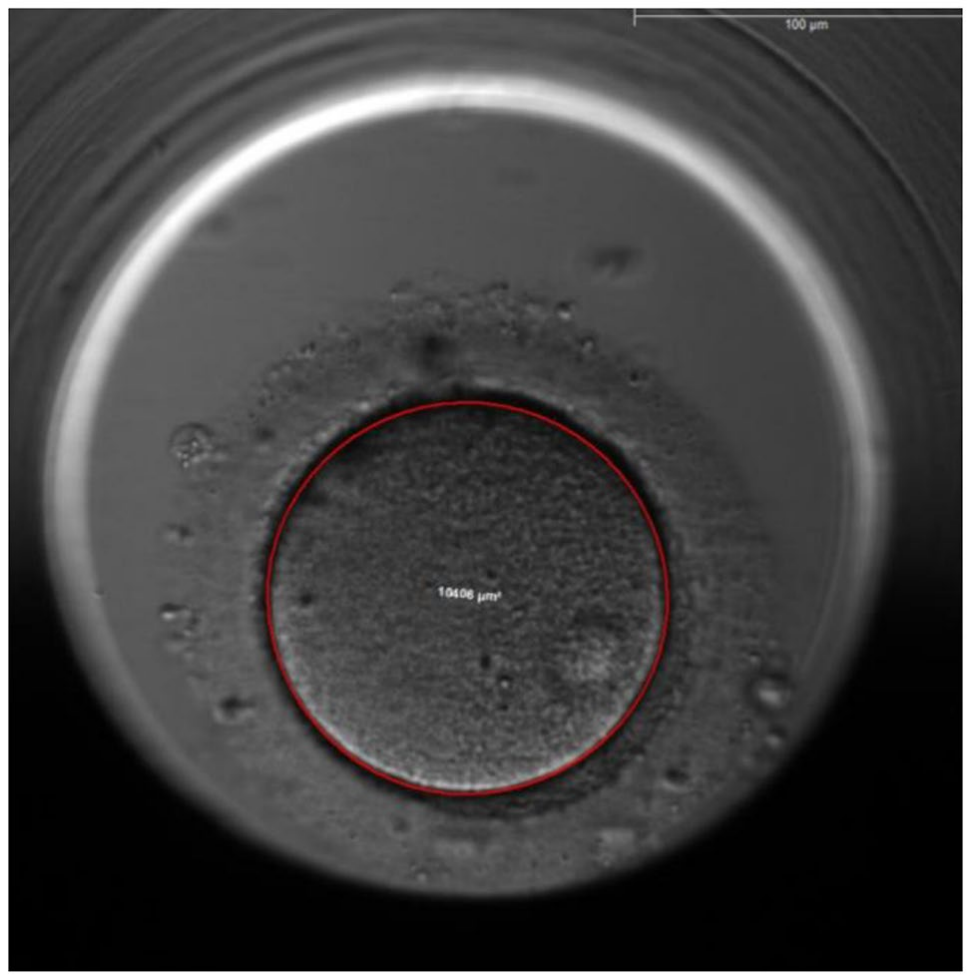
Measurement of ooplasm area by ellipse tool immediately after ICSI

**Fig. 4:**
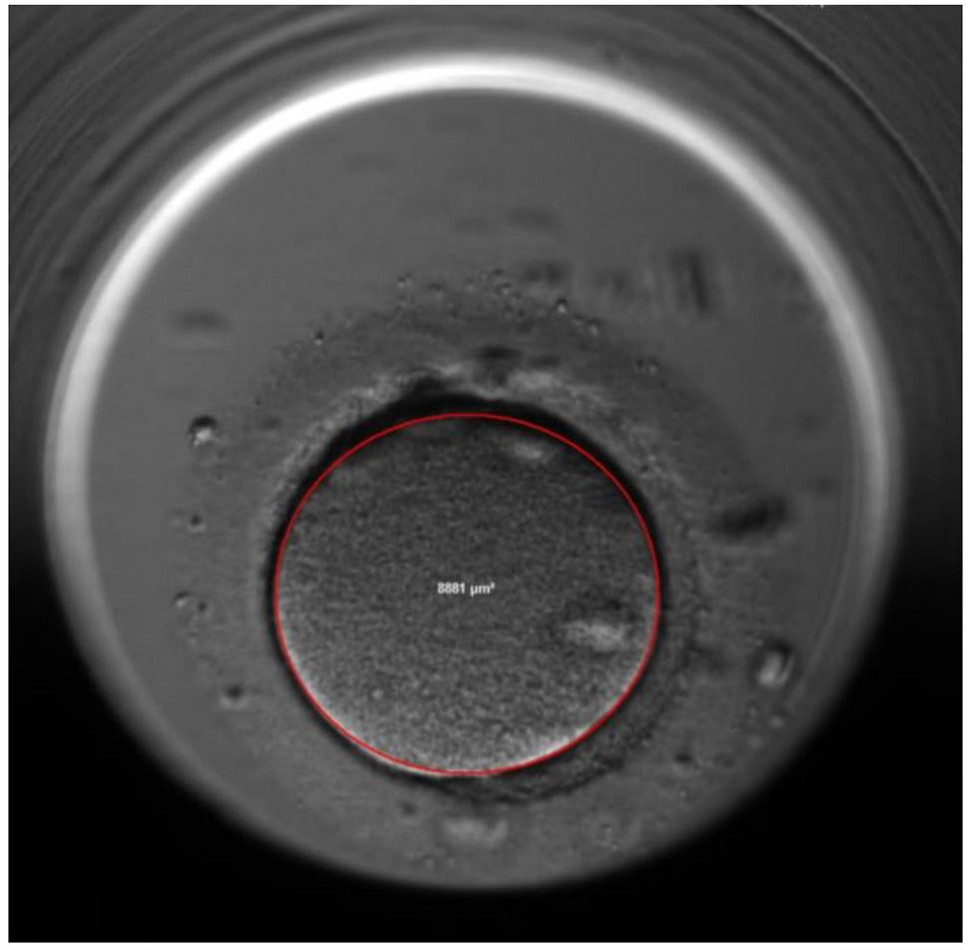
Measurment of the oplasm after 2PN fading, the same oocyte of Fig. 3

The area and volume of the ooplasm was calculated using the ellipse tool of the EmbryoViewer™, the area is calculated automatically by this tool (Figs. 3 and 4). The volume of the ooplasm of each oocyte was measured every 1 h after ICSI till before first cleavage. The reproducibility of the measurement was confirmed by repeating the ooplasm measurements 20 times for 10 oocytes (Table 3).

**Table 3:** Reproducibility of measurements of ooplasm area

Oocyte	Mean µm^2^	SD µm^2^	CV (%)
1	10553.75	55.58	0.53%
2	10786.2	48.38	0.45%
3	9873	54.05	0.55%
4	10349.55	43.24	0.42%
5	11288.65	54.66	0.48%
6	11133.65	65.39	0.59%
7	10306.2	57.08	0.55%
8	10072.55	57.72	0.57%
9	10542.75	42.17	0.40%
10	9977.7	59.42	0.60%

**Fig. 5:**
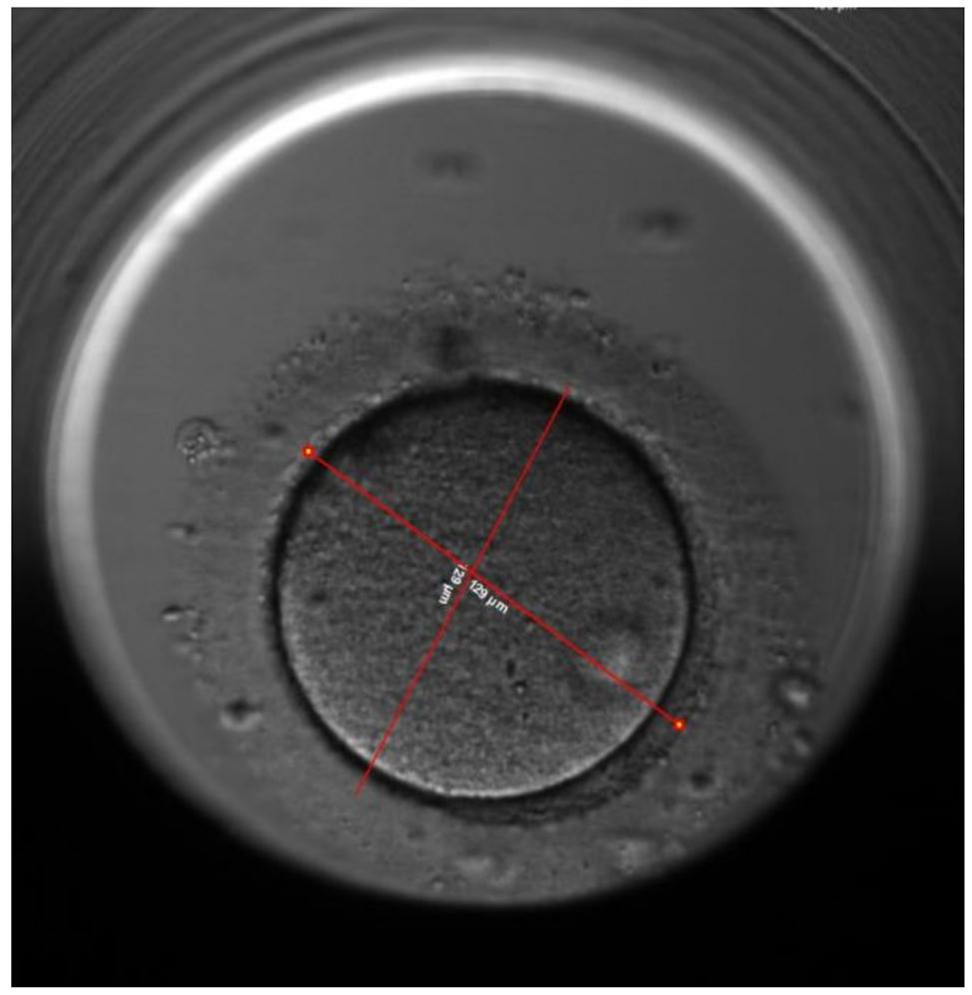
Measurement of oocyte diameter

The internal diameter of the oocyte was measured using the distance function of the EmbryoViewer™ software (Fig. 5). The reproducibility of the measurement was confirmed by repeating the ooplasm measurements 20 times for 10 oocytes (Table 4).

**Table 4:** Reproducibility measurement of oocyte diameter

Oocyte	Mean µm	SD µm	CV (%)
1	126.18	0.82	0.65%
2	122.85	1.18	0.96%
3	128.55	1.23	0.96%
4	130.11	0.72	0.55%
5	129.02	0.90	0.70%
6	122.55	1.00	0.81%
7	128.55	1.15	0.89%
8	130.25	1.07	0.82%
9	121.55	1.15	0.94%
10	129.70	1.17	0.91%

### Calculations

The measured area of the ooplasm was used to calculate the radius (r) of the ooplasm using the general formula of circles r = $\sqrt{\frac{A}{\pi }}$ [29], where r is the radius, A is the area and π equals to 3.14.

The volume of the ooplasm was calculated using the formula for calculating the volume of a sphere, that is, v_1_ = $\frac{4}{3}$π r^3^ [29], where v_1_ is the volume of the ooplasm.

RC: Regression coefficient. SE: Standard error. LCI: Lower confidence interval.

UCI: Upper confidence interval.

The volume of the oocyte was calculated from the measured diameter (d) by dividing the diameter over 2 to get the radius r using the equation r = $\frac{d}{2}$, then using the r to calculate the volume of the oocyte v_2_$=\frac{4}{3}$ π r^3^.

The volume of the perivitelline (P_v_) space was calculated by subtraction of the volume of the ooplasm from the volume of the oocyte using the formula P_v_ = v_2_ – v_1_.

### Morphokinetics

The embryo morphokinetics were checked from ICSI time till the 8-cell stage. The precise times of each embryo division and the developmental parameters were determined in hours post-ICSI (hpi). The following are the definitions of the developmental markers that were utilised in this part of the study: time of emergence of second polar body (tPB2), time of appearance of the pronuclei (tPNa), time of pronuclei fading (tPNf), division into 2 cells (t2), division into 3 cells (t3), division into 4 cells (t4), and division into 5 cells (t5). The duration of the second cell cycle (CC2), also known as the amount of time it takes for 2 cells to 3 cells. The time that elapses between the division of a 3-blastomere embryo and a 4-blastomere embryo is referred to as the second synchronisation (S2) or (t4-t3). The time duration of the third cell cycle is known as CC3 (t5-t3) and the interval between 2 and 5 cells is the variable t5-t2 which combines the concepts of cell synchrony and cell cycle. The division into the eight-cell stage (t8).

### Statistical analysis

Data were analysed using the multiple regression analysis for correlation. A two-tailed *p*-value less than 0.05 (p < 0.05) was considered significant and all data analyses were performed using Microsoft Excel 2010 (Microsoft Corporation, USA). The ROC and AUC tests were performed using SAS (SAS Institute, USA).

## Results

This study has demonstrated that there is a correlation between the three measured morphometric characteristics and embryo implantation. The first characteristic is the change in the average ZP thickness that decreased by 32.7% ± 5.3% at 70 h for the implanted embryos (Group 1) versus 23.6% ± 4.8% for non-implanted embryos (Group 2) p = 0.000. Figures 1 and 2 show the measurements of the ZP of the same implanted embryo with different thickness. The picture in Fig. 1 was taken immediately after ICSI whereas the picture in Fig. 2 was taken at 70 h before embryo transfer, the two figures show that there is difference in the thickness of the ZP.

The second characteristic is the average reduction in the volume of the ooplasm which is 20.5% ± 4.3% in Group 1 versus 15.1% ± 5.2% in Group 2, p = 0.000. Figures 3 and 4.

The third characteristic is the increase in the volume of the perivitelline space which was 38.1% ± 7.6% in Group 1 versus 31.6% ± 9.7% in Group 2 p = 0.000. The changes in morphometric parameters are summarized in Table 5.

**Table 5:** Values of the multiple regression analysis

Variable	RE	SE	P value	LCI	UCI
Change in ZP thickness	-6.101	0.195	0.000	-6.484	-5.719
Change in ooplasm volume	-1.795	0.216	0.000	-2.183	-1.335
Change in PV space volume	0.098	0.016	0.000	0.066	0.129

The results of multiple regression analyses revealed regression coefficients (RCs), standard errors (SEs), P values (P < 0.05) and lower and upper 95% confidence intervals (LCI and UCI) for the morphometric embryo variables, with significant coefficients for Group 1 (100% implantation) and Group 2 (0% implantation) are presented in Table 6 which shows significant regression coefficients for the changes in ZP thinning, reduction in ooplasm volume and increase in the volume of the PV space.

**Table 6:** Change in Morphometric characteristics

Parameter	Group 1, Pregnant	Group 2, Non-Pregnant	P-value
Decrease in ZP Thickness SD	32.7% 5.3%	23.6% 4.8%	0.000
Decrease in ooplasm volume SD	20.5% 4.3%	15.1% 5.2%	0.000
Increase in PV space volume SD	38.1% 7.6%	31.6% 9.7%	0.000

The morphokinetic parameters of implanted versus non-implanted embryos were as follows: tPB2 = 4.1 ± 2.1 vs. 4.4 ± 1.7 hpi with p > 0.05, tPNa = 10.5 ± 3.1 vs. 10.8 ± 3.3 hpi with p > 0.05, tPNf = 24.3 ± 4.5 vs. 24.9 ± 4.9 hpi with p > 0.05, t2 = 28.1 ± 6.9 vs. 29.7 ± 8.1 with p > 0.05, t3 = 38.1 ± 6.2 vs. 38.9 ± 7.9 with p > 0.05, t4 = 41.1 ± 7.1 vs. 40.8 ± 7.6 hpi with p > 0.05, t5 = 51.3 ± 8.5 vs. 50.2 ± 9.2 hpi with p > 0.05, CC2 = 10.0 ± 4.1 vs. 9.2 ± 4.7 hpi with p > 0.05, CC3 = 13.2 ± 5.3 vs. 11.3 ± 6.5 hpi with p > 0.05, S2 = 3.0 ± 3.1 vs. 1.9 ± 3.9 hpi with p > 0.05, t5-t2 = 23.2 ± 7.2 vs. 20.5 ± 7.9 hpi with p > 0.05, t8 = 59.4 ± 8.9 vs. 57.2 ± 7.6 hpi with p > 0.05, tSC = 85.8 ± 9.1 vs. 85.8 ± 7.4 hpi with p > 0.05, tIB = 96.0 ± 6.6 vs. 96.9 ± 6.4 hpi with p > 0.05, tB = 102.6 ± 7.1 vs. 104.1 ± 6.8 hpi with p > 0.05, tEB = 110.8 ± 8.0 vs. 111.1 ± 7.2 hpi with p > 0.05. The summary of the morphokinetic parameters of implanted versus non-implanted embryos is summarised in Table 7.

**Table 7:** Morphokinetics of implanted versus non-implanted embryos

Parameter	Implanted embryos	Non implanted embryos	p
tPB2	4.1 ± 2.1	4.4 ± 1.7	p > 0.05
tPNa	10.5 ± 3.1	10.8 ± 3.3	p > 0.05
tPNf	24.3 ± 4.5	24.9 ± 4.9	p > 0.05
t2	28.1 ± 6.9	29.7 ± 8.1	p > 0.05
t3	38.1 ± 6.2	38.9 ± 7.9	p > 0.05
t4	41.1 ± 7.1	40.8 ± 7.6	p > 0.05
t5	51.3 ± 8.5	50.2 ± 9.2	p > 0.05
CC2	10.0 ± 4.1	9.2 ± 4.7	p > 0.05
CC3	13.2 ± 5.3	11.3 ± 6.5	p > 0.05
S2	3.0 ± 3.1	1.9 ± 3.9	p > 0.05
t5-t2	23.2 ± 7.2	20.5 ± 7.9	p > 0.05
t8	59.4 ± 8.9	57.2 ± 7.6	p > 0.05

**Table 8:** Abnormal embryo cleavage

Abnormal cleavage	Percentage
Total percent of abnormal cleavage	16.3%
1 to 3 cell stage	5.2%
2 to 4 cell stage	3.7%
3 to 5 cell stage	3.2%
4 to 6 cell stage	1.2%
Others	3.0%

**Fig. 6:**
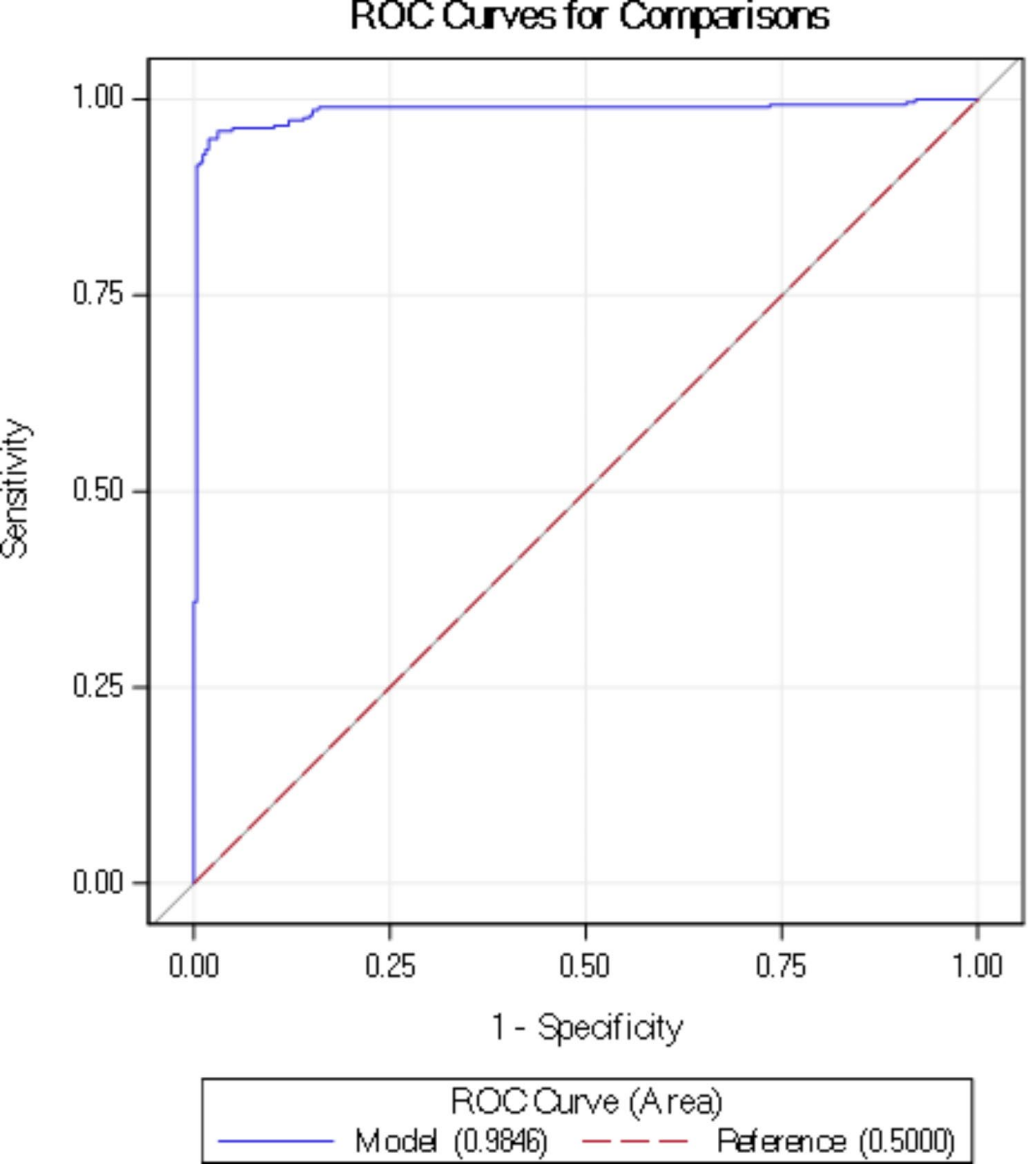
ROC curve

The ROC statistics show an AUC value of 0.9846, a standard error of 0.00568 and a confidence interval between 0.9735 and 0.9958, these values are presented in Table 8; Fig. 6.

The included embryos in this study were acceptable at the morphologic and morphokinetics level. On the other hand, 16.3% of the embryos displayed abnormal cleavage, such as direct cleavage from the stage of 1 to 3 cells and from the stage of 2 to 4 cells. The details of abnormally cleaved embryos are presented in Table 9. The 16.3% of embryos that exhibited abnormal division were not included in this study.

**Table 9:** ROC statistics

Area Under Curve	Standard Error	Confidence Interval	
0.9846	0.00568	0.9735	0.9958

## Discussion

There is a consensus amongst embryologist on the morphologic features of top-quality embryo on day 3. However, there is no consensus or strong evidence on the implantation potential of the top-quality D3 embryo [22]. This necessitates investigation to elucidate and formulate characteristics and features of the embryo that is likely confer it the potential for implantation [30–33].

The routine embryo scoring systems do not permit accurate timing of embryo morphometry or morphokinetics. The advent of time lapse incubators permits accurate observation of embryo development and facilitate the development of morphokinetic and morphometric algorithms to choose embryo with best implantation capability [34].

The features offered by time lapse videography can be linked to implanted embryos and thus provide new opportunities for enhancing embryo scoring [18, 35] thereby enabling the creation of objective tools for the selection of viable embryo(s).

Previous studies showed that embryo morphometric features were linked to embryo implantation [36, 37], but this prediction was not decisive in the identification of the viable embryo [38].

This study investigated three morphometric embryonic characteristics for selecting embryos with implantation potential on addition to the existing and well-established morphological and morphokinetic embryo scoring algorithms. The first characteristic of those investigated in this study is the percentage of the thinning of the ZP, the second is the percent of reduction in the ooplasm volume and the third is the percent increase in the volume of the perivitelline space.

Our results indicate that the implanted embryos in this study were characterised by the following features: (i) higher percentage of thinning of the ZP at 72 h, (ii) higher percentage of reduction in the volume of the ooplasm at the timethe pronuclei fades and (iii) higher percentage of increase in the volume of the perivitelline space at the time the pronuclei fades.

Our findings related to the changes in zona pellucida thickness matches previous studies that investigated the thickness of the zona pellucida [24, 39] and the effect of zona pellucida thickness on embryo implantation [40, 41].

The present investigation revealed that the proportion reduction in the thickness of the ZP that may confer the potential for implantation in human day 3 embryos, the negative value of the RC demonstrates that an inverse relationship exists between the percentage thinning of the ZP and potential for embryo implantation, which indicates that the higher percentage of the ZP thinning, the higher is potential for implantation.

The reduction in the volume of ooplasm for implanted embryos in this study has a, negative value of the RC which indicates the inverse relationship that exists between ooplasm reduction and implantation, indicating that the higher the percentage of ooplasm reduction the higher the potential for embryo implantation.

The increase in the volume of the perivitelline space for embryos that implanted in the present study, has a positive value of the RC which indicates the existence of a direct relationship between the increase in the perivitelline space and the potential for embryo implantation.

The AUC value is 0.9846 which indicates a strong correlation between the studied characteristics of the embryos and the implantation potential.

To the best of our knowledge this is the first report that links three embryonic developmental characteristics to its potential for implantation. This novel methodology for the identification of potentially viable day 3 embryos developed by the present investigation currently requires manual calculations that consumed, about 1.5 h for each embryo, after which the measurements were transferred manually to an excel sheet for further analysis. However, the time taken for calculation can be dramatically reduced to a few seconds if the calculation is automated. The investigators are of the opinion this methodology can be incorporated not only in the embryoscope, but also in embryo image analysis software. The latter could prove useful and economical for laboratories that could not afford expensive time lapse incubators because the image analysis system will enable work with an inverted microscopes in laboratories that do not own embryoscope. Indeed, investigations on the morphometric development of the human embryo was attempted successfully in the late 1990’s using the Saturn™ embryo image analysis system by one of us (JA). The authors are convinced that the novel morphometric methodologies developed and described in the present study can be performed in the absence of an embryoscope using an appropriately modified image analysis software system. A patent lodgement for the present findings is being processed.

It appears plausible the morphometric methodologies developed in this study for the identification of potentially viable human day 3 embryos could significantly reduce the subjectivity that has mired the selection of human embryo(s) for transfer in ART treatment procedures.

The incorporation of the present morphometric methodologies in the embryoscope will enable the performance of the embryonic morphometric measurements on the images and videos of the embryos stored in the incubator thereby circumventing the need to take the embryos out of the incubator, which could expose the embryo to the adverse effects induced by the alterations in the ambient temperature, pH, humidity and light stress which is an advantage offered by the embryoscope.

The points of strength of this study are it has the potential to reduce the subjectivity of present embryo scoring methods by allowing images to be graded straight from embryo photographs or videos. The features used in this study allow more objective embryo scoring.

It allows for the development of a suitable standard that can be used for extrapolation based on the results of all of the scoring systems that have been that have been achieved in this study. The limitation of the study is that it is time consuming and it takes 1.5 h to perform the measurements for one embryo, it can be implemented routinely after a software is developed to perform the measurements automatically. It needs more development to study more features until the blastocyst stage.

The study is being continued in the laboratories of YA and JA to determine the morphometric developmental characteristics of the embryo, especially on the blastomeres, ZP, PVS, and other embryonic features from day 0 through day 7 of human preimplantation development. The investigations are anticipated to arrive at an algorithm that could interphase with an artificial intelligence module for enhanced and more objective embryo scoring and less invasive procedure for the selection of embryos with the potential for implantation in ART treatment.

## Conclusion

According to the findings of this study, the implanted embryos exhibited three morphometric characteristics that are additional to the known morphological and morphokinetical characteristics. The three new characteristics are higher percentage of the zona pellucida becoming thinner at 72 h, higher percentage of a reduction in the volume of the ooplasm at the time the pronuclei fade and a higher percentage of an increase in the volume of the perivitelline space at the time the pronuclei fade.

## Data Availability

The datasets used or analyzed during the current study are available from the corresponding author on reasonable request.

## Abbreviations

2PN: Two Pronuclei
A: Area
ART: Assisted Reproduction Technology
AUC: Area Under Curve
CC: Cell Cycle
d: Diameter
ET: Embryo Transfer
ICSI: Intracytoplasmic Sperm Injection
IVF: In Vitro Fertilisation
LCI: Lower Confidence Interval
OR: Odd Ratio
P: Perivitelline
PVS: Perivitelline Space
Q: Quartile
r: Radius
RC: Regression Coefficient
ROC: Receiver Operating Characteristics
UCI: Upper Confidence Interval
v: Volume
ZP: Zona Pellucida
ZPTV: Zona Pellucida Thickness Variation
